# Secular trends in functional abilities, health and psychological well-being among community-dwelling 75- to 95-year-old cohorts over three decades in Helsinki, Finland

**DOI:** 10.1177/14034948211007688

**Published:** 2021-04-25

**Authors:** Hanna R. Öhman, Helena Karppinen, Tuuli E. Lehti, Mia T. Knuutila, Reijo Tilvis, Timo Strandberg, Hannu Kautiainen, Kaisu H. Pitkala

**Affiliations:** 1 Geriatric Medicine, University of Helsinki and Helsinki University Hospital, Finland; 2 Department of General Practice and Primary Health Care, University of Helsinki, Finland; 3 Social Services and Health Care, City of Helsinki, Finland

**Keywords:** Physical functioning, well-being, self-rated health, community dwelling, cohort comparison

## Abstract

*Background:* Life expectancy has increased markedly in the past decades. Thus, it is of great importance to understand how people are ageing and if the trajectories of health and disability are changing over time. This study aimed to examine trends in functional abilities and health in independent cohorts of people aged 75–95 over three decades. *Methods:* This Helsinki Ageing Study consists of repeated cross-sectional postal surveys examining independent cohorts of old people (75, 80, 85 and 90+ years old). This study combined data from four waves (1989, 1999, 2009 and 2019). *Results:* In the most recent wave, there was an increase in the portion of participants who were able to walk outdoors easily (75-year-olds *p*=0.03, 80-year-olds *p*=0.002, 85-year-olds *p*<0.001; *p* for linearity for the study year effect, all adjusted for sex). Fewer people in the youngest age group (75-year-olds) needed daily help from another person in 2019 compared to the earlier waves (*p*=0.02 for linearity for the study year). Over the past three decades, the proportions of self-reported good mobility have risen 8.7% (95% confidence interval (CI) 2.3–15.1) in 75-year-olds, 11.7% (95% CI 3.9–19.6) in 80-year-olds and 20.1% (95% CI 10.7–29.4) in 85-year-olds, after adjusting for sex. Furthermore, in 2019, more people rated their health as good and scored better in psychological well-being than in the previous waves among 75-, 80- and 85-year-olds. However, no improvements were found among 90+-year-olds in any of these variables. **Conclusions: People between 75 and 85 years old are presently feeling and functioning better than their predecessors. This may be an important objective for both economics and health policy.**

## Introduction

Life expectancy has increased rapidly during the last century. In many developed countries, older adults – generally referring to those aged ⩾65 years – are the fastest-growing part of the population [1]. As this age group is also the most predisposed to chronic illnesses and disabilities, it is valuable to understand how the health and functioning of an ageing population develops over time. Will the added years in lifespan be healthy and functional or merely a struggle, with increased morbidity and disability? The individual and public importance of this issue has stimulated great research activity around the subject [2].

Physical activity and functional abilities are important in preserving independence and good quality of life. Many epidemiological studies during the past decades have shown that disability rates were declining in recent cohorts [3–6]. Christiansen et al. studied two independent cohorts of people aged >90 years and found out that the cognitive abilities as well as the activities in daily living were better in the more recent cohort [3]. The same trend has also been detected in younger cohorts of older adults: in a Finnish study of people aged 65–69 years [4] and in a Swedish study of 75-year-olds [5]. However, there have been contradictory findings, especially during the last years, as some studies found no decline or even increasing trends of disability [2,7–10]. In the previous wave of the Helsinki Ageing Study (2009), this negative phenomenon was also detected in participants aged 75–85 when independent cohorts of older home-dwelling people living in Helsinki in 1989, 1999 and 2009 were studied [10]. According to the studies reporting unfavourable results, while older adults with multiple diseases and diagnosis in the most recent cohorts may survive longer because of modern medicine and more active medical care, on the other hand, they may live their final years with more frailty and disability [2,9].

Health and functioning go hand in hand in old age, as many diseases lead to disability and loss of independence. Co-morbidities are difficult to compare between cohorts, since diagnosing diseases has become more active in old age, and new diagnoses such as dementia and osteoporosis have emerged during the last decades. Self-rated health (SRH) is thought to reflect the comprehensive picture of health as perceived by the individual, including medical diagnoses, health conditions, symptoms, functional disabilities and psychosocial symptoms. This widely used health indicator is considered to be reliable [11]. SRH has been shown to be a significant predictor of health outcomes, including mortality, morbidity and functional independence [12]. Poorer SRH is also associated with a greater use of health care [13]. Despite the important role of SRH, there is a scarcity of studies evaluating the temporal trends of SRH.

Well-being and health are closely related, and the connection becomes even more important as the prevalence of chronic illness increases with advancing age [14]. Psychological well-being (PWB) and a person’s perspective of their own health status play a role in how they maintain their functional independence and cope with the difficulties of ageing [15]. A growing body of research literature suggests that PWB may even be a protective factor in health, reducing the risk of chronic physical illness and disability later in life. PWB is considered an important dimension of quality of life [14].

This study examines the trends in functional abilities, health and PWB in independent cohorts of people aged 75–95 years over three decades.

## Methods

### Study participants

The present study combines data from four waves of the Helsinki Ageing Study. The details of this study have been described in previous papers [10,16].

Cross-sectional random samples of older people were mailed surveys in Helsinki, Finland. The 1989 sample included people 75, 80 and 85 years old, whereas in 1999, 2009 and 2019, additional random samples of 90- and 95-year-olds were included. Here, we combined the 90- and 95-year-olds’ samples.

The response rates for 1989, 1999, 2009 and 2019 were 93% (*n*=660), 80% (*n*=2598), 73% (*n*=1637) and 74% (*n*=1758), respectively. The response rates are based on estimates of how many survey recipients may have died, moved away or been institutionalised between the most recent Helsinki population census (half a year behind) and mailing the questionnaires.

All cohorts were assessed by surveys that used the same design and identical questions. Random samples of older people within these age groups were recruited from the Finnish National Population Register. Respondents living in long-term care facilities were excluded from the study. One reminder was sent to those who did not initially respond. All studies have been approved by the Helsinki University Hospital Ethics committee.

### Questionnaire

The questionnaire included questions on socio-demographic status (education, living conditions, marital status), clinical diagnoses, functional abilities and difficulties in daily life, PWB and SRH.

To assess the functional abilities, we asked the participants whether they were able to walk outdoors (easily/with difficulties or not at all) and whether they needed another person’s help daily (yes/no). Self-reported mobility disability has been shown to be a reasonably good substitute for an objective measure of mobility in observational studies [17].

A total of 20 common medical diagnoses were listed, and participants were asked to give a yes/no answer to each. The number of diagnoses reported were counted and the Charlson Comorbidity Index was calculated accordingly [18]. Respondents evaluated their health on a four-point scale as ‘healthy’, ‘quite healthy’, ‘quite unhealthy’ or ‘unhealthy’. The first two items were classified as good SRH and the other two as poor SRH.

We used six questions to evaluate participants’ PWB [19]. We inquired about (a) life satisfaction (yes/no), (b) feeling useful (yes/no), (c) having plans for the future (yes/no), (d) having zest for life (yes/no), (e) feeling depressed (seldom or never/sometimes/often or always) and (f) suffering from loneliness (seldom or never/sometimes/often or always). The responses ‘yes’ to questions (a)–(d) and ‘seldom or never’ to questions (e) or (f) added one point each to the total sum, ‘sometimes’ to questions (e) or (f) added 0.5 point each and ‘no’ to questions (a)–(d) and ‘often or always’ to questions (e) or (f) gave 0 points. We then created a PWB score by summing the ratings from the six questions and dividing the sum by the number of questions the participant had answered. The total score ranged from 0 to 1, with higher scores indicating better PWB.

### Data analyses

Data are presented as means with standard deviation (*SD*) or as counts (*n*) with percentages (%). Hypothesis of linearity were tested using the Cochran–Armitage test, logistic models and analysis of variance with an appropriate contrast. Models included sex as covariates when appropriate. Tests of interactions between sex, age and cohort were carried out by including a product term in each logistic regression model. The bootstrap method was used when the theoretical distribution of the test statistics was unknown or when assumptions were violated (e.g. non-normality). The normality of variables was evaluated graphically and by using the Shapiro–Wilk W test. All analyses were performed with Stata v16.1 (StataCorp LP, College Station, TX).

## Results

Table I presents the demographic and basic health information of the participants in independent cohorts I–IV (1989, 1999, 2009 and 2019). The percentage of men among participants increased gradually from 27% in 1989 to 37% in 2019. In 1989, 74% of the participants had less than eight years of formal education, while in 2019 that dropped to 25%. Widowhood was also less common in the later cohorts.

**Table I.** Demographic characteristics of the people participating in the four waves of the Helsinki Ageing Study.

	I (1989) *N*=660	II (1999) *N*=2598	III (2009) *N*=1637	IV (2019) *N*=1758	*p* for linearity
Women, *n* (%)	484 (73)	1851 (71)	1126 (69)	1128 (64)	<0.001
Age (years), *n* (%)					<0.001
75	245 (37)	735 (28)	399 (24)	424 (24)	
80	220 (33)	716 (28)	393 (24)	420 (24)	
85	195 (30)	643 (25)	357 (22)	394 (22)	
90–95	0 (0)	504 (19)	488 (30)	520 (30)	
Widowed, *n* (%)	291 (45)	1179 (47)	681 (42)	604 (36)	<0.001
Education <8 years (%)	427 (74)	1337 (54)	603 (37)	417 (25)	<0.001
CCI, *M* (*SD*)	1.4 (1.3)	2.1 (1.9)	2.0 (1.8)	1.7 (1.6)	0.023

CCI: Charlson Comorbidity Index [18]; *SD*: standard deviation.

**Figure 1.**
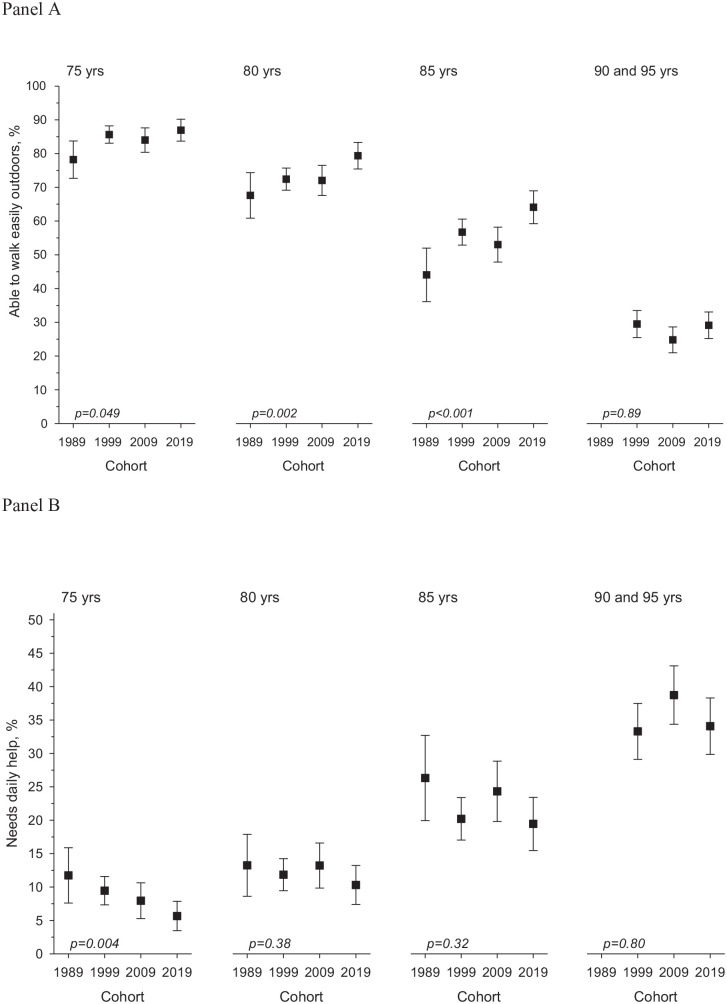
(a) Ability to walk easily outdoors and (b) need for daily help among the participants of the Helsinki Ageing Study in 1989, 1999, 2009 and 2019 in 75-, 80-, 85- and 90+-year-old age groups (proportions with 95% confidence intervals; *p* for linearity, adjusted for sex). In (b), the scale is reversed so that smaller percentages indicate positive changes.

The proportion of participants being able to walk easily outdoors and who did not need daily help increased between 1989 and 1999. In the 2009 cohort, the positive trend seemed to level off. However, in the most recent cohort, the portion of participants who reported being able to walk easily outdoors increased again (75-year-olds *p*=0.03, 80-year-olds *p*=0.002, 85-year-olds *p*<0.00, *p* for linearity for the study year effect, all adjusted for sex). Moreover, fewer people in the youngest age group (75-years-old) needed daily help from another person in 2019 compared to the earlier cohorts (*p*=0.02 for linearity for the study year). However, among the 90+-year-olds, the functioning did not show significant changes over the three decades (Figure 1). We further explored whether an increasing proportion of males modified the cohort effect. There was no interaction in any of the age groups concerning being able to walk easily outdoors between sex and cohort. The same applies to age groups 80, 85 and 90 concerning the need for daily help. However, there was an interaction in the 75-year-old age group between sex and cohort (*p*=0.030). There was no interaction between age and cohort concerning being able to walk easily outdoors or need for daily help.

Over the four study waves of the Helsinki Ageing Study, the proportion of participants reporting the ability to walk easily outdoors has increased in 75-year-olds by 8.7% (95% confidence interval (CI) 2.3–15.1), in 80-year-olds by 11.7% (95% CI 3.9–19.6) and in 85-year-olds by 20.1% (95% CI 10.7–29.4), all adjusted for sex. The need for daily help has decreased by –6.1% (95% CI –10.8 to –1.4) in the 75-year-olds, by –2.9% (95% CI –8.4 to +2.5) in the 80-year-olds and by –6.9% (95% CI –14.4 to + 0.7) in the 85-year-olds, all adjusted for sex.

**Figure 2.**
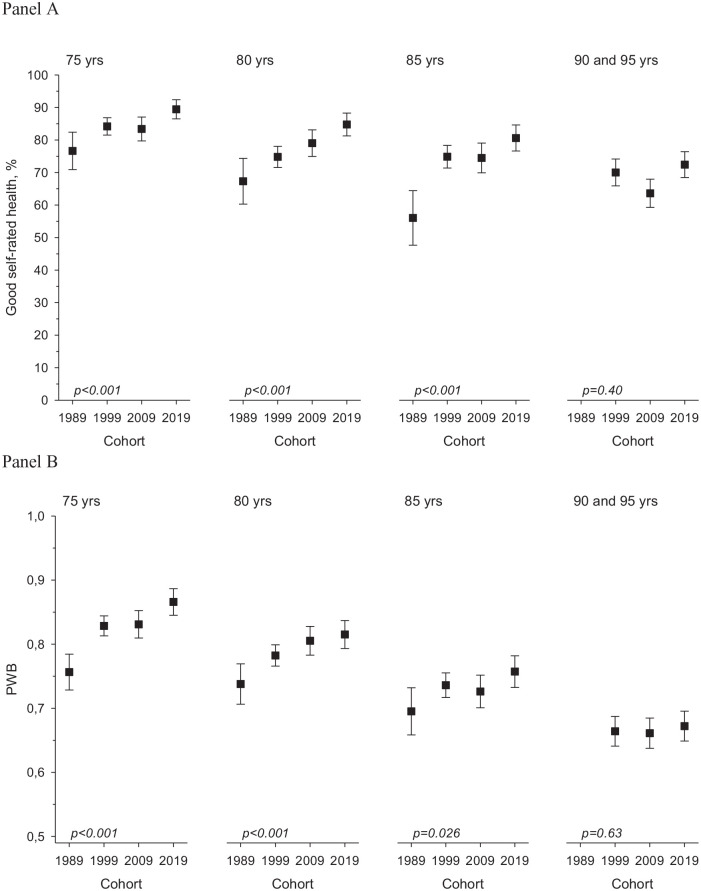
(a) Good self-rated health (proportions with 95% confidence intervals). (b) Good psychological well-being (PWB; means with standard deviations) among the participants of the Helsinki Ageing Study in 1989, 1999, 2009 and 2019 in 75-, 80-, 85- and 90+-year-old age groups (*p* for linearity, adjusted for sex).

The Charlson Comorbidity Index has decreased slightly since 1999, and this also applies to the latest study wave and to the 90- to 95-year-olds. Most of the participants in all ages and all cohorts rated their health as good. The proportion of 75-, 80- and 85-year-olds reporting good SRH has increased with every subsequent cohort (Figure 2).

PWB showed the clearest improvement in the younger age cohorts over the study years (75-year-olds *p*<0.001, 80-year-olds *p*<0.001, 85-year-olds *p*=0.021; *p* for linearity for the study year effect, adjusted for sex). Significant differences in PWB for the 90- and 95-year-olds were not noted (Figure 2).

## Discussion

In this study, we surveyed four independent cohorts of community-dwelling people aged 75–95 years old every 10 years for three decades. Our findings showed improvement in functional abilities, PWB and SRH – especially in the 75-, 80- and 85-year-olds – whereas the oldest age group (90+-year-olds) remained stable. There was also a decreasing trend in the Charlson Comorbidity Index in all aged cohorts. The unfavourable trends in disability seen in the previous wave of the Helsinki Ageing Study [10] seem to be improving according to the latest cohort.

The positive changes in health and functioning were most evident in the youngest age groups. This may be due to improved and more active health care, early diagnostics and management of risk factors such as hypertension, hyperlipidaemia and smoking [20]. An increasing number of male participants may contribute to the better functional abilities, as previous studies indicate that both prevalence and incidence of disability seem to be higher for women than for men [21]. However, our analyses were adjusted for sex, thus implying that the improved functional abilities may be a true finding. As disability can also be defined as the discrepancy between an individual’s capacity and environmental demand, the recent improvements in housing standards, advances in technology and social policy may also be factors facilitating more active and independent life, despite age-related limitations in function [22].

The oldest age group (90+) did not show any significant changes in functional abilities or other health assessments. It may be that the inevitable physiological deterioration in this very advanced age group cannot be overcome with improved medical care or favourable changes in the environment and society [23].

In the latest cohorts, nearly 10% more 75- to 85-year-olds were able to walk easily outdoors, and about 5% less needed another person’s daily help. These proportions are large at the population level and may have significant implications for the lower need for services. However, the study population represents urban, community-dwelling older adults, and thus the results may not be generalisable to the whole population.

Our findings related to the functional abilities, especially of the youngest aged groups, are generally consistent with those of several previous studies [4–6,24]. Falk et al. studied two birth cohorts of Swedish 75-year-olds in 1976–1977 and 2005–2006 and reported that the later cohorts were more independent in activities of daily living (ADLs) and more engaged in leisure activities than the earlier cohorts [5]. Physical abilities of two independent cohorts of same-aged Europeans (2004–2005 and 2013) showed improvement in ADLs and instrumental ADLs in those aged ⩾70 years. However, this positive trend was only seen among participants from Northern Europe (Sweden and Denmark). According to the authors, this may be due to the more positive economic development in Northern Europe than Southern Europe during the study period [24]. This can also partly explain the favourable changes in the functional abilities in our study. However, several studies from the USA have also shown contradictory findings concerning disabilities [2,8,9].

Concerning functional abilities, our 90+ cohorts suggest contradictory findings compared to some recent studies [3,25,26]. A Danish study compared two 90+ cohorts born 10 years apart and suggested that disability was decreasing in the later cohort [3]. Similar findings were reported in a Chinese study that compared three groups of Chinese older adults born 10 years apart, aged 80–89 years, 90–99 years or 100–105 years at the time of surveys, which were performed in 1998 or 2008. In the later cohort, ADL disability had significantly decreased [25]. The Tampere Vitality 90+ Study from Finland suggested a similar trend over 18 years from 2001 to 2018 [26]. However, in both the Chinese and Danish studies, the performance in ADLs improved more than the actual physical performance tests. This may reflect the rapid changes in the living standards and advances in the use of assisting technology in daily life among older people [3,25].

In our study, PWB and SRH also seemed to be improving in the latest cohort. This is a positive sign, as standards of ‘good health’ seem to change over time, and higher expectations concerning one’s health status may affect the ratings, even if the objective health status is improving [27]. Improvements in social status (fewer widowers and more years of education) might partly explain the favourable trends in well-being assessments.

Our results of improved SRH are in line with some other studies on this topic [28,29]. A Swedish study examining three different cohorts of women from 1992 to 2017 reported a positive trend in the SRH of those who were 70 years old in the recent cohort [28]. A longitudinal study from Norway also found an increasing rate of higher SRH scores in older age groups [29].

Older people report more diseases and health problems than younger age groups. At the same time, they seem to cope fairly well with many of the activities of independent living and regard their health as good. This may reflect their ability to adapt to health-related problems as well as other challenges in life [7].

A major strength of this study is that it is based on representative samples of community-dwelling older people examined with identical questionnaires over a 30-year period. The instruments used in the questionnaire were well validated, and the response rate was good. This is an exceptionally long study comparing independent cohorts in their functioning, health and well-being over 30 years. Furthermore, to our knowledge, this is one of the few studies simultaneously comparing functioning, health and well-being.

Several limitations should be kept in mind when considering the findings of this study. The participants were community-dwellers living in an urban area, and therefore the results cannot be generalised to other cohorts. Moreover, SRH and PWB may have different implications in various social and cultural settings [12]. Although the response rate in this sample is relatively high, there is a slight decline compared to the previous study waves. The study excluded institutionalised individuals, which may be a confounding factor, as the threshold between the community and institution changes over time. The proportion admitted to long-term care facilities has decreased significantly over the three decades of the study period, and those residing in institutional care in Finland are far more disabled than those 10–30 years ago [30]. Thus, there are relatively more people with disabilities living in their own homes. Therefore, the favourable difference in functioning and well-being may be an underestimate of the true difference between the cohorts. The participants were also volunteers. However, the same bias applied for all the cohorts.

To conclude, this study provides positive evidence of improving functional abilities, health and well-being of older adults today aged 75–85 years old. This may have positive implications for economics and health policy, as the need for social services such as home care and nursing home beds is reduced. Moreover, the improving health and well-being may imply that the older people stay active and a productive part of society for longer.
